# Pro-Environmental Behaviors: Relationship With Nature Visits, Connectedness to Nature and Physical Activity

**DOI:** 10.1177/08901171221119089

**Published:** 2022-08-11

**Authors:** Andreia Teixeira, Ronaldo Gabriel, José Martinho, Mário Santos, Aurélio Faria, Irene Oliveira, Helena Moreira

**Affiliations:** 1 56066 University of Trás-os-Montes and Alto Douro, Vila Real, Portugal; 2 Centre for the Research and Technology of Agro-Environmental and Biological Sciences (CITAB), Department of Sports Science, Exercise and Health, 56066 University of Trás-os-Montes and Alto Douro, Vila Real, Portugal; 3 Geosciences Centre (CGeo), Department of Geology, 56066 University of Trás-os-Montes and Alto Douro, Vila Real, Portugal; 4 Centre for the Research and Technology of Agro-Environmental and Biological Sciences (CITAB), Laboratory of Applied Ecology, 56066 University of Trás-os-Montes and Alto Douro, Vila Real, Portugal; 5 Center in Sports Sciences, Health Sciences and Human Development (CIDESD), Department of Sport Science, 56056 University of Beira Interior, Covilhã, Portugal; 6 Centre for the Research and Technology of Agro-Environmental and Biological Sciences (CITAB), Department of Mathematics, 56066 University of Trás-os-Montes e Alto Douro, Vila Real, Portugal; 7 Center for Computational and Stochastic Mathematics, CEMAT-IST-UL, University of Lisbon, Lisbon, Portugal; 8 Research Center in Sports Sciences, Health Sciences and Human Development (CIDESD), Centre for the Research and Technology of Agro-Environmental and Biological Sciences (CITAB) Department of Sports, Exercise and Health Sciences, 56066 University of Trás-os-Montes e Alto Douro, Vila Real, Portugal

**Keywords:** sustainable behaviors, visits to natural environments, emotional connectedness to nature, accelerometer, gender differences, environmental health, health policies

## Abstract

**Purpose:**

Examine the association of visits to the natural environment, connectedness to nature, physical activity, and the adoption of pro-environmental behaviors (PEBs) in individuals aged 18 years or older.

**Design:**

Cross-sectional study.

**Setting:**

City of Vila Real, located in the north of Portugal.

**Subjects:**

We recruited 194 individuals (61 men and 133 women) aged 18-75 years.

**Mesures:**

A self-administered questionnaire was used to measure nature visits, connectedness to nature, PEBs, and demographic characteristics. Neighborhood green space was appreciated through a Simplified Land Occupation Map and physical activity was measured using ActiGraph accelerometers (wGT3X-BT).

**Analysis:**

Correlations and nonlinear canonical correlation analysis were used to analyze the data. The coefficients of canonical and multiple correlations were calculated.

**Results:**

Nature visits were associated with involvement in environmental volunteering (V = .317, P ≤ .05) among men. In these, higher levels of moderate-vigorous PA were associated with green travel behavior (η^2^ = .325, P ≤ .05). Connectedness with nature was related (P ≤ .05) to private sphere behaviors, such as purchase of eco-products (η2 = .191) and local/seasonal products (η2 = .186) in females and encouraging care and protection of natural environment (η2 = .336, P ≤ .01) in males.

**Conclusions:**

Nature visits, connection to nature, and physical activity levels were related to the adoption of PEBs in the private and public sphere, and these relationships differed between men and women.

## Introduction

Individual behavior is widely recognized as a major contributor to numerous environmental problems, including environmental pollution and biodiversity loss;^1^ and therefore, the adoption of pro-environmental behaviors (PEBs) is essential for the development of more sustainable societies.^2^

Pro-environmental behavior refers to the adoption of actions aimed at minimizing environmental harm or actively restoring the natural environment, which can be carried out in the private domain (eg, recycling, green purchase, saving water and energy) or public domain (eg, encouraging others to care for and protect the environment, belonging to an environmental group).^3,4^

A growing body of evidence suggests that exposure to natural environments (eg, urban green space, forest, grassland, wetlands, cropland, pastureland, lakes, rivers, and seas) increases the adoption of sustainable behaviors^5,6^ and improves health and well-being.^7-10^ The study conducted by Alcock, White, Pahl, Duarte-Davidson and Fleming^11^ demonstrated that greater exposure to nature contributes to a stronger probability of individuals adopting environmentally friendly behaviors, such as recycling, green-travel, buying environmentally friendly products, and volunteering for environmental projects. Martin, White, Hunt, Richardson, Pahl and Burt^5^ also found that visiting natural spaces at least once a week was associated with a higher propensity to engage in household PEBs such as recycling and buying ecological products.

There is an increasing realization that natural contact alone may be insufficient to accrue these potential benefits to pro-environmentalism. Previous studies have reported that the pathways that explain this relationship include biocentric values,^12^ place attachment,^13^ psychological restoration,^14^ and connectedness to nature.^15-17^ Recently, two systematic reviews^15,17^ have demonstrated that connection with the natural environment trigger greater involvement in self-reported pro-environmental behavior, particularly energy and water conservation, anti-consumerism, pro-environmental political activism and financial support for environmental organizations. Defined by Mayer and Frantz^18^ as the “experiential sense of oneness with the natural world,” connection to nature generates greater respect, admiration, and responsibility for the conservation of the natural environment, encouraging the appropriate use of its resources and the preservation of species and natural habitats.^19^

The role of physical activity in the adoption of environmentally sustainable behaviors requires further research. To our knowledge, only the study developed by Fang et al^20^ and conducted in children has explored the relationship between physical activity and the adoption of PEBs. Physical activity is not only a health behavior but also a valuable tool for climate change mitigation,^21,22^ enabling the achievement of many of the 2030 Sustainable Development Goals (SDGs),^23^ such as good health and well-being (SDG 3), gender equity (SDG 5), reducing inequalities (SDG 10), sustainable cities and communities (SDG 11), and climate action (SDG 13).^22^ According to Abu-Omar et al^21^ there are several interconnections between PA promotion and climate change mitigation, related to active transportation, use of green spaces and recreational or exercise facilities. Recently, Salvo et al^22^ demonstrated that climate mitigation may benefit from multiple PA promotion strategies, largely due to shifts toward more active forms of travel and recreation. Thus, creating structures that support walking or cycling for commuting rather than using the car reduces greenhouse gas emissions and noise pollution, improves air quality, and offers opportunities to create healthier and more sustainable cities, contributing to climate change mitigation.^24-26^

The literature also lacks studies that investigate the influence of certain sociodemographic variables, such as the number of children and dog ownership on pro-environmental behavior. Despite the attention that studies have focused on various domains of the private sphere of environmental behavior (eg, recycling, saving water and energy), little attention has been given to behavior related to the public sphere, such as encouraging others to be pro-environmental, environmental volunteering, and environmental organization membership. Finally, few studies have conducted separate analyses in men and women.^27-29^

The aim of this research was to analyze, across both genders, the relationship between visits to the natural environment, connectedness to nature, and physical activity and the adoption of pro-environmental behaviors in individuals aged 18 years or older.

## Methods

### Study Location

**Figure 1.**
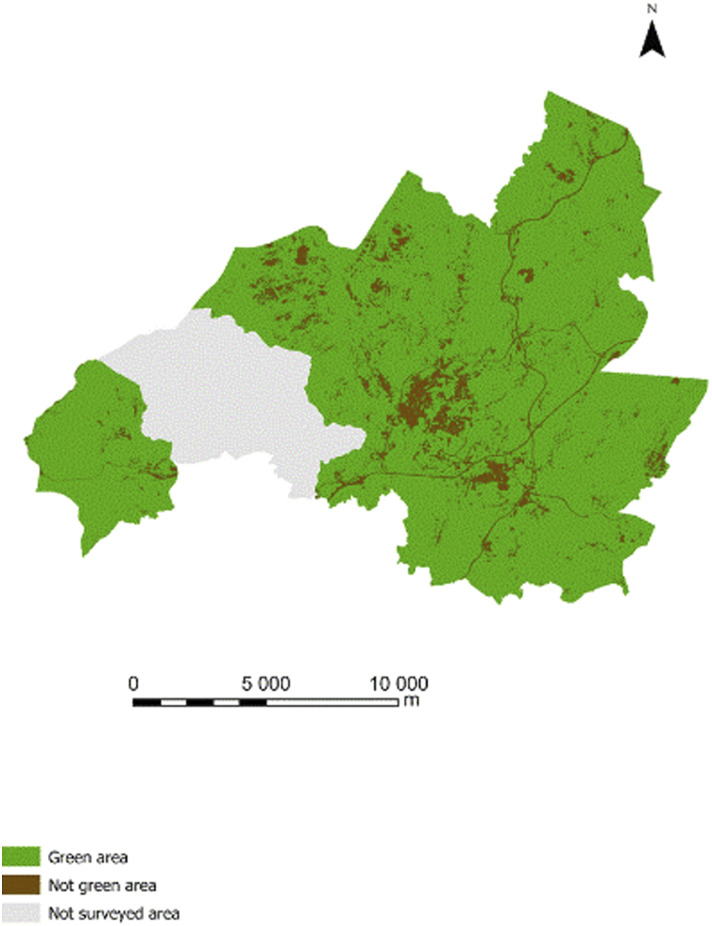
Study location.

This study was conducted in Vila Real, a city located in the north of Portugal. Based on place of residence, our sample comprised 14 parishes in this municipality,^30^ covering 220.58 km^2^ with a human population of approximately 44 644 inhabitants. These parishes benefit from 20 240 ha of green space, a ratio of 4534 m^2^ of green space^31^ per inhabitant (Figure 1).

### Study Design and Sample

We conducted a cross-sectional study between December 2020 to February 2021. The sample included 61 men (41.42 ± 15.37 years) and 133 women (39.93 ± 15.60 years), aged 18 to 75 years old. Participants were recruited through advertising via e-mail and by posters. The posters were disseminated on various social networks (eg, Twitter) and the emails were addressed to individuals in a laboratory database where the research was conducted. In turn, some of these emails were also provided by individuals who came to complete the assessments. After the assessment sessions, all participants had access to the results and were informed about them. The delivery of the results acted as a reward to the participants for being part of the study and on the other hand, constituted a focus for recruitment of the sample. No financial or other incentives were given to the participants to integrate the study. The eligibility criteria included (1) age ≥18 years, (2) ability to read and understand Portuguese, and (3) willingness to wear an activity monitoring device on the wrist for four consecutive days. Participants who did not complete all evaluations or did not use the accelerometer for a minimum of 10 hours per day^32,33^ on four assessment days were excluded from the sample. The participants provided verbal and written consent. Each individual authorized the publication of information and images inherent exclusively to the data collection. The evaluators were trained, both technically and scientifically.

### Procedures

All participants were individually tested in a laboratory. First, the individuals provided written consent to participate in the study. Each participant completed the questionnaire composed of questions related to nature visits, adoption of pro-environmental behaviors, and demographic variables. They also completed the Connectedness to Nature Scale.^18^ After that, the researchers explained and delivered the accelerometer. They provided written information on how to use the sensor, as well as a phone contact for clarification of any questions. The laboratory evaluations took about 20 minutes. After the period of using the accelerometer, the participants returned to the lab to deliver the device. All data collection was conducted by two of the researchers and several team meetings were held to ensure the quality of the predefined methodological procedures.

### Measures

#### Green space coverage

Green and non-green areas were categorized through a Simplified Land Occupation Map.^34^ Green areas resulted from the grouping of three typological categories: agriculture, forests, bushes and spontaneous herbaceous vegetation. The non-green areas included the following categories: artificialised, non-vegetated surfaces, wetlands and water surfaces. The geographical significance of green and non-green areas by place of residence was calculated using the areas of previously reported categories for each place of residence relative to the total area of residence, expressed as a percentage. The Land Simplified Occupation Map resolution was 10 m, which means that the minimum cartographic unit was 100 square meters.

#### Connectedness to Nature Scale

The CNS is designed to explicitly measure the degree to which a person feels emotionally connected to nature.^18^ In this study, we used the Portuguese version of the CNS.^35^ The scale is composed of 14 items with response options on a 5-point Likert scale ranging from 1 (strongly disagree) to 5 (strongly agree) and three items were reverse-scored (4,12 and 14). Higher scores indicate that one feels a greater connection to nature. The scale has previously been demonstrated to have a Cronbach alpha of .84.^18^ In the current sample, the alpha was .7 indicating acceptable reliability. The cut-off point for CNS was 3.71 points, with the objective of having an equal number of observations in each group.

#### Nature visits

The nature visit frequency measure used ordinal categorical responses to the question “Thinking about the last seven days, how often, on average, have you spent your leisure time out of doors (eg, parks, woods, beaches, forests, gardens or similar)?” The response options were scored as follows: 0-4: (0) “never”; (1) “once a week”; (2) “2 to 3 times per week”; (3) “4 to 5 times per week”; and (4) “> 5 times per week.”, responses adapted from the Alcock et al^11^

#### Pro-environmental behaviors

Self-reports on seven specific behaviors (with Yes/No responses) were applied and included the following categories: recycling (“I usually recycle items rather than throw them away), buying eco-friendly (“I usually buy eco-friendly products and brands”), buying seasonal/local products (“ I usually buy seasonal or locally grown food”), walking/cycling for short journeys (“I choose to walk or cycle instead of using my car when I can”), encouraging others to be pro-environmental (“I encourage other people to protect the environment”); environmental volunteering (“I volunteer to help care for the environment”) and environmental organization membership (“I am a member of an environmental or conservation organization”).^11^

#### Physical Activity

Triaxial accelerometers wGT3X-BT (Actigraph, Pensacola, Florida, USA) were used to measure the PA of the participants who were worn on the non-dominant wrist. It has been reported that this device was the most commonly used in clinical and epidemiological research.^36^ Participants were instructed to wear the accelerometer for four days^37^ (two weekdays and two weekends), at all times except when engaging in water-based activities such as swimming or showering. The device was programmed for 6 am on the first day of evaluation, and the PA records were considered for 15-second periods and initialized to capture and store accelerations at 100 Hz.^37^ For the accelerometer data to be acceptable, a minimum period of 10 h was considered^32^ and the non-wear time considered was that developed by Choi et al^33^ Accelerometer data processing and analysis were conducted using ActiLife (Version 6) software. The variables evaluated by accelerometry were as follows: total PA (TPA, min/week), moderate-vigorous PA (MVPA, min/week), and steps/day (n^º^). A cutoff point of 4836 counts per minute was used to assess minutes in moderate-vigorous.^38^ The cut-off points for MVPA and steps days were 150 min/week^39^ and 10 000 steps/day.^40,41^

#### Demographic information

Participants answered questions regarding age, gender, marital status, number of children, education, occupational status and dog ownership. The operationalization of these variables is provided in the Supplementary Material (Table S1).

### Statistical Methods

Descriptive analysis was performed to show the characteristics of the sample. Continuous data were expressed as mean ± standard deviation, and qualitative variables were presented as absolute frequencies and percentages. The normality of the distributions was analyzed using the Kolmogorov-Smirnov test. Student’s t-test or the Mann-Whitney test for data with asymmetric distribution were used to compare groups. The associations between variables were determined using the Pearson correlation coefficient for continuous scale variables when data were normally assumed or Spearman correlation when at least one variable was ordinal or when scale data were non-normal.^42^ For an independent variable categorical and a dependent variable of scale or interval level, the Eta value was obtained and used as a measure of association. The square of Eta was interpreted as the proportion of variation in the dependent variable explained by the independent variable.^43^ However this statistical procedure does not allow us to infer whether the sign of the association.^42^ Thereafter, we tried to develop binary logistic regressions as well as neural networks to predict pro-environmental behaviors; however, the models showed no predictive power and were therefore discarded. Due to that, nonlinear canonical correlation analysis (OVERALLS)^44^ was used, as an exploratory multivariate technique, to assess the relative contributions of k>2 sets of data, demographic and variables (nature visits, connectedness nature, and physical activity). This technique allows to determine how similar the sets of categorical variables are to one another as described by Frie and Janssen.^45^ We used 13 variables classified into three sets: (1) pro-environmental behaviors, (2) nature visits, connectedness to nature and physical activity, and (3) demographic variables. The labels of the sets, variables and categories in the data and the symbols that represent the categories in the graphics are given in Table S2. To simplify the interpretation of the results, centroid plots were developed for the two genders separately. For each PEB, tables with percentages of dimensions 2 and 3 are presented (Table S3 and Table S4), highlighting the significant associations identified in the pre-analysis. All models were stratified by gender and statistical significance was assumed at P < .05. All analyses were carried out using IBM SPSS, version 27.0 (Chicago, IL, USA).

## Results

### Data Description

The mean age of the sample was 40.09 ± 15.49 years. No statistically significant differences were identified between the two genders in relation to levels of PA, green space coverage, PEBs, and connectedness to nature (Table 1). The mean MVPA values identified for each gender were within the values recommended in literature. Men documented a higher number of weekly visits to natural spaces (P ≤ .01) than women.

**Table 1.** Baseline Characteristics of Study Participants.

Variables	Overall (n= 194)	Men (n= 61)	Women (n=133)	P
	Mean ± SD	Mean ± SD	Mean ± SD	
Age (years)	40.09 ± 15.49	41.42 ± 15.37	39.93 ± 15.60	.93 ^a^
Physical activity (PA)				
Total PA (min/week)	703.16 ± 299.84	659.16 ± 265.99	723.35 ± 313.03	.23 ^a^
Moderate-vigorous PA (min/week)	178.03 ± 115.96	182.75 ± 110.24	175.86 ± 118.84	.50 ^a^
Steps/day (n^º^)	12 768.54 ± 3802.02	13 166.23 ± 3843.36	12 586.14 ± 3783.46	.33 ^b^
Pro-environmental behaviors (n^º^)	4.51 ± 1.23	4.49 ± 1.41	4.52 ± 1.15	.83 ^a^
Nature visits (times/week)	1.30 ± 1.06	1.54 ± 1.03	1.19 ± 1.05	< .01^a^
Connectedness to nature (points)	3.73 ± .43	3.68 ± .44	3.76 ± .43	.31 ^a^
Green space coverage (%)				
Green area	71.51 ± 15.15	69.43 ± 14.60	72.47 ± 15.36	.22 ^a^
Not-green area	28.49 ± 15.15	30.57 ± 14.60	27.53 ± 15.46	.22 ^a^

Abbreviation: SD, standard deviation; PA, physical activity

^a^Mann-Whitney test.

^b^T-test for independent samples.

### Demographic Data

Most participants were female (68.6%), aged 49 years or less (71.2%), married or cohabiting (47.4%), had no children (47.9%), and more than half (63.9%) were employed (Table 2). Regarding educational level, 41.2% of the individuals declared that they had completed high school, followed by 33.5% who claimed to have a graduate, master’s or doctorate degree. In our sample, 30.4% visited natural environments 2-3 times a week and owned a dog. Approximately half of the sample (50.5%) met the recommended levels of PA (MVPA>150 min/week) and 75.3% of individuals performed 10 000 steps per day. Regarding PEBs, more than 70% of individuals said they actively engage in behaviors related to recycling, buying environmentally friendly products and brands, and buying seasonal or locally grown food. This positive involvement was also expressed in relation to making travel more environmentally friendly and encouraging others to care for and protect their environment. Only 42.3% of the participants said they volunteered to help and care for the environment, and only 4.1% belonged to an environmental or nature conservation organization.

**Table 2.** Demographic Data, Pro-Environmental Behaviors, and Nature Visits.

Variables	Overall (n = 194)	Men (n = 61)	Women (n = 133)
	n (%)	n (%)	n (%)
Age groups			
<33 years	75 (38.7)	22 (36.1)	53 (39.8)
33-49 years	63 (32.5)	21 (34.4)	42 (31.6)
≥50 years	56 (28.9)	18 (29.5)	38 (28.6)
Marital status			
Single	89 (45.9)	26 (42.6)	63 (47.4)
Married/cohabiting	92 (47.4)	35 (57.4)	57 (42.9)
Separated/divorced/widowed	13 (6.7)	—	13 (9.8)
Number of children			
0	93 (47.9)	29 (47.5)	64 (48.1)
1	37 (19.1)	7 (11.5)	30 (22.6)
2	54 (27.8)	22 (36.1)	32 (24.1)
≥3	10 (5.2)	3 (4.9)	7 (5.3)
Education			
Elementary school	5 (2.6)	1 (1,6)	4 (3.0)
Middle school	10 (5.2)	5 (8.2)	5 (3.8)
High school	80 (41.2)	24 (39.3)	56 (42.1)
University	34 (17.5)	10 (16.4)	24 (18.0)
Post-graduation/master’s/doctorate	65 (33.5)	21 (34.4)	44 (33.1)
Occupational status			
Employee	124 (63.9)	40 (65.6)	84 (63.2)
Unemployed	4 (2.1)	2 (3.3)	2 (1.5)
Retired	3 (1.5)	—	3 (2.3)
Student	55 (28.4)	17 (27.9)	38 (28.6)
Other	8 (4.1)	2 (3.3)	6 (4.5)
Owns a dog			
Non	135 (69.6)	42 (68.9)	93 (69.9)
Yes	59 (30.4)	19 (31.1)	40 (30.1)
Nature visits			
Never or once a week	126 (64.9)	30 (49.2)	96 (72.2)
2 to 3 times per week	59 (30.4)	29 (47.5)	30 (22.6)
≥4 times per week	9 (4.6)	2 (3.3)	7 (5.3)
Connectedness to nature			
≤3 .71 points	98 (50.5)	32 (52.5)	66 (49.6)
>3.71 points	96 (49.5)	29 (47.5)	67 (50.4)
Moderate-vigorous physical activity			
<150 min/week	96 (49.5)	29 (47.5)	67 (50.4)
≥150 min/week	98 (50.5)	32 (52.5)	66 (49.6)
Steps/day			
<10 000 steps/day	48 (24.7)	10 (16.4)	38 (28.6)
≥10 000 steps/day	146 (75.3)	51 (83.6)	95 (71.4)
Pro-environmental behaviors			
Recycling			
Not	35 (18.0)	16 (26.2)	19 (14.3)
Yes	159 (82.0)	45 (73.8)	114 (85.7)
Eco-products			
Not	50 (25.8)	21 (34.4)	29 (21.8)
Yes	144 (74.2)	40 (65.6)	104 (78.2)
Seasonal/local			
Not	19 (9.8)	8 (13.1)	11 (8.3)
Yes	175 (90.2)	53 (86.9)	122 (91.7)
Green travel			
Not	54 (27.8)	11 (18.0)	43 (32.3)
Yes	140 (72.2)	50 (82.0)	90 (67.7)
Volunteering			
Not	112 (57.7)	31 (50.8)	81 (60.9)
Yes	82 (42.3)	30 (49.2)	52 (39.1)
Encouragement			
Not	26 (13.4)	8 (13.1)	18 (13.5)
Yes	168 (86.6)	53 (86.9)	115 (86.5)
Membership			
Not	186 (95.9)	57 (93.4)	129 (97.0)
Yes	8 (4.1)	4 (6.6)	4 (3.0)

### Pro-Environmental Behaviors: Relationship With Other Analyzed Variables

In both genders, age, marital status and occupational status showed a significant association (P ≤ .05) with connectedness to nature (Table 3). In men, this relationship with the natural environment was also related (P ≤ .01) to dog ownership (η^2^ = .342) and green space coverage (η^2^ = .426). In women, this bond was associated with the number of visits to nature (*r*_s_ = .233, P ≤ .01).

**Table 3.** Associations Between Connectedness to Nature, Pro-Environmental Behaviors, and the Visits to Natural Spaces With Other Variables.

Variables	All (n = 194)			Men (n = 61)			Women (n= 133)		
	Connectedness to nature (points)	Pro-environmental behaviors (n^º^)	Nature visits (times/week)	Connectedness to nature (points)	Pro-environmental behaviors (n^º^)	Nature visits (times/week)	Connectedness to nature (points)	Pro-environmental behaviors (n^º^)	Nature visits (times/week)
Physical activity (PA)									
Total PA (min/week)	.109 *r*	.113 *r*	.058 *r*_s_	.021 *r*	.270* *r*	.194 *r*_s_	.133 *r*	.040 *r*	.026 *r*_s_
Moderate-vigorous PA (min/week)	.038 *r*	.074 *r*	.061 *r*_s_	−.019 *r*	.272* *r*	.210 *r*_s_	.061 *r*	−.028 *r*	−.018 *r*_s_
Steps/day (n^º^)	.170* *r*	.177* *r*	.181* *r*_s_	.138 *r*	.232 *r*	.287* *r*_s_	.189* *r*	.149 *r*	.118 *r*_s_
Socio-demographics data									
Age (years)	.283*** *r*	.130 *r*	.121 *r*_s_	.303* *r*	.015 *r*	.032 *r*_s_	.277** *r*	.194* *r*	.180* *r*_s_
Marital status	.314*** η^2^	.136 η^2^	.200** *V*	.384** η^2^	.066 η^2^	.226* *V*	.293** η^2^	.170 η^2^	.159 *V*
Number of children (n^º^)	.126 *r*	.098 *r*	.121 *r*_s_	.136 *r*	.086 *r*	.046 *r*_s_	.126 *r*	.106 *r*	.150 *r*_s_
Education	−.072 *r*_s_	.070 *r*_s_	.026 *r*_s_	−.145 *r*_s_	.053 *r*_s_	.012 *r*_s_	−.038 *r*_s_	.084 *r*_s_	.035 *r*_s_
Occupational status	.255* η^2^	.224* η^2^	.141 *V*	.414* η^2^	.138 η^2^	.174 *V*	.268* η^2^	.295* η^2^	.096 *V*
Owns a dog	.089 η^2^	.025 η^2^	.214* *V*	.342** η^2^	.270* η^2^	.140 *V*	.031 η^2^	.114 η^2^	.432** *V*
Green area (%)	.198** *r*	.135 *r*	.127 *r*_s_	.426** *r*	.104 *r*	.236 *r*_s_	.107 *r*	.152 *r*	.106 *r*_s_
Nature visits (times/week)	.197** *r*_s_	.189** *r*_s_	—	.176 *r*_s_	.213 *r*_s_	—	.223** *r*_s_	.170 *r*_s_	—
Connectedness to nature (points)	—	.168* *r*	.197** *r*_s_	—	.214 *r*	.176 *r*_s_	—	.148 *r*	.223** *r*_s_

Abbreviation: *r*, Pearson correlation coefficient; η^2^, Eta square, *r*_s_, Spearman’s correlation coefficient; V, Cramér’s V coefficient

Note: *P ≤ .05; **P ≤ .01; ***P ≤.001.

In men, pro-environmental behaviors showed a significant association (P ≤ .05) with total and moderate-vigorous physical activity (r = .270 and r = .272, respectively) and dog ownership (η^2^ = .270). However, in women, the adoption of sustainable behaviors was related (P ≤ .05) to age (r = .194) and occupational status (η^2^ = .295).

Our results also revealed that, in men, visits to natural spaces were related (P ≤ .05) to the number of daily steps (*r*_s_ = .287), marital status (*V* = .226), and in women to age (*r*_s_ = .180) and dog ownership (*V* = .432).

The correlations developed for each item on the list of pro-environmental behaviors are presented in Table 4. There were no significant associations in the two genders for the item “membership.”

**Table 4.** Associations Between Each Pro-Environmental Behavior and the Demographic Variables, Nature Visits, Connectedness to Nature, Green Area, and Physical Activity.

Variables	Age	Marital status	Number of children	Education	Occupational status	Owns a dog	Nature visits	Connectedness to nature	Green area	Moderate-vigorous PA	Steps/day
*Men (*n = *61)*	η^2^	*V*	η^2^	*V*	*V*	*V*	*V*	η^2^	η^2^	η^2^	η^2^
Recycling	.260*	.164	.210	.315	.331	.079	.110	.086	.020	.153	.243
Eco-products	.157	.136	.114	.251	.163	.264*	.211	.137	.048	.125	.070
Seasonal/local	.031	.058	.006	.273	.123	.156	.127	.100	.070	.108	.124
Green travel	.143	.059	.118	.198	.197	.039	.226	.008	.050	.325*	.201
Volunteering	.106	.014	.081	.253	.187	.117	.317*	.075	.082	.135	.081
Encouragement	.216	.254*	.185	.145	.205	.261*	.117	.336**	.018	.074	.104
Membership	.098	.094	.135	.236	.076	.035	.049	.098	.097	.121	.011
*Women (*n*=133)*											
Recycling	.267**	.264**	.212*	.252	.268*	.199*	.067	.110	.007	.010	.031
Eco-products	.334***	.302**	.287**	.129	.340**	.048	.198	.191*	.123	.058	.137
Seasonal/local	.080	.016	.043	.138	.132	.060	.057	.186*	.139	.071	.024
Green travel	.026	.130	.064	.186	.127	.069	.148	.046	.042	.034	.166
Volunteering	.093	.107	.161	.141	.243	.008	.090	.062	.105	.020	.020
Encouragement	.045	.033	.128	.101	.170	.022	.153	.008	.114	.039	.067
Membership	.064	.105	.025	.162	.058	.020	.109	.035	.065	.018	.010

Abbreviation: PA, Physical activity; η^2^, Eta square; V, Cramér’s V coefficient.

Note: *P ≤ .05; **P ≤ .01; *** P ≤.001.

### Private and Public Dimensions of Pro-environmental Behaviors

#### Recycling

**Figure 2.**
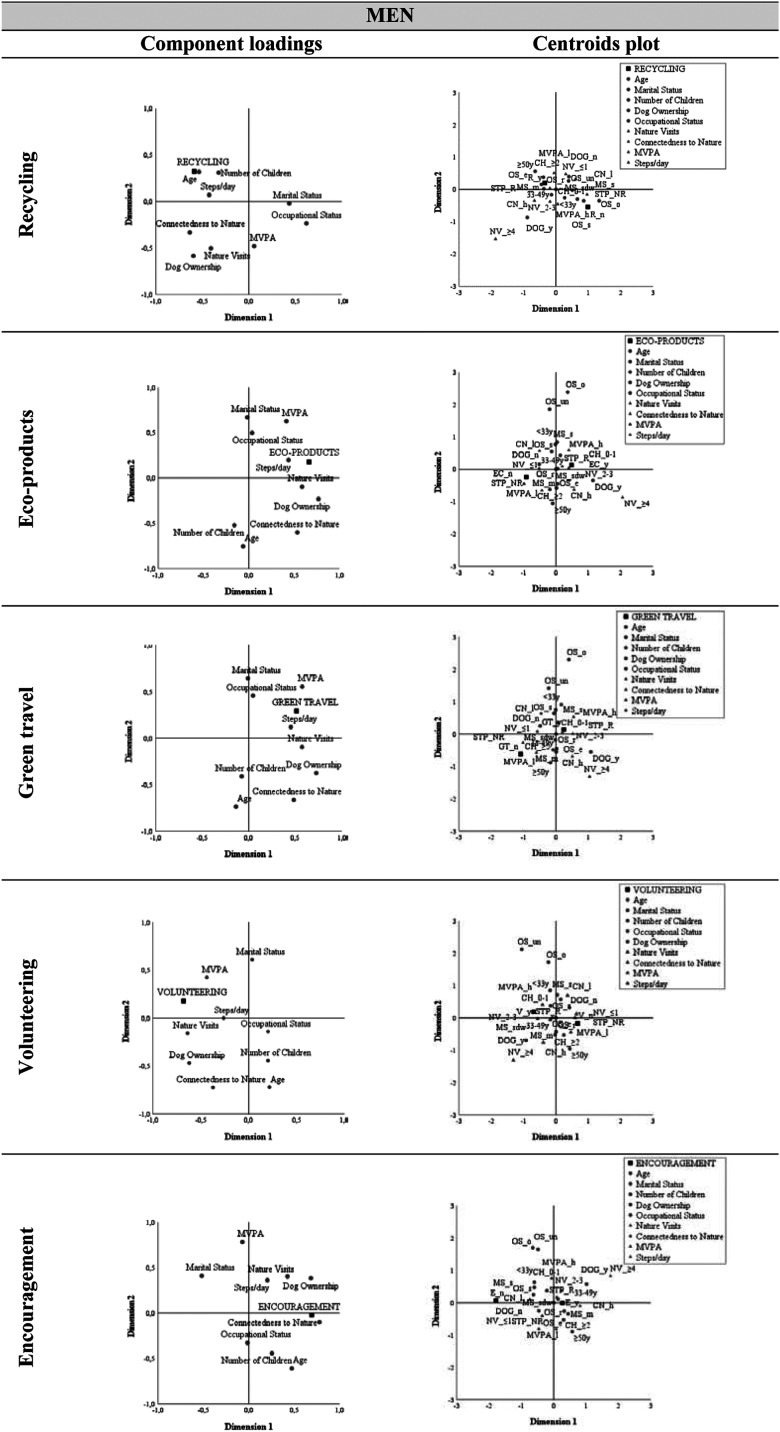
Component loadings and centroids plot in men; (please see Table S2).

**Figure 3.**
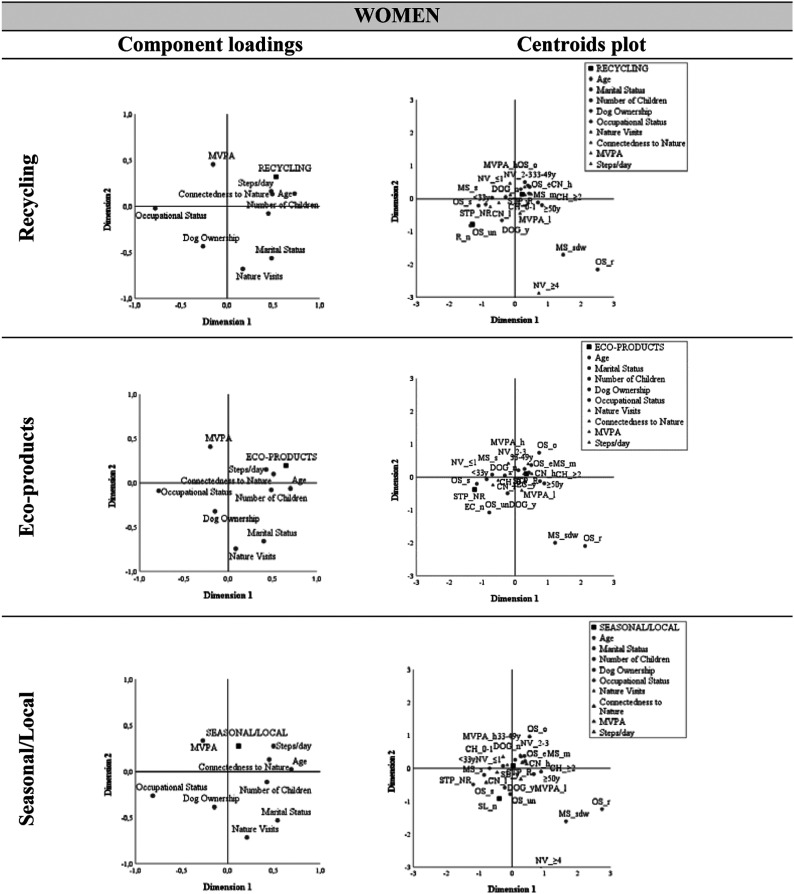
Component loadings and centroids plot in women; (please see Table S2).

In both genders, greater involvement with recycling was observed in the older age groups, namely in the 33-49 age group (81.0% in men and 92.9% in women), but particularly in the 50 and older age group (83.3% and 94.7%, respectively) (Figures 2 and 3; Tables S2 and S3). In women, it is also noteworthy that employed, married, and those who did not own a dog were those who documented recycling more. Although the association between the number of children and recycling was only significant in women (Table 4), it was identified in both genders that individuals with at least two children adopted more recycling (84.0% in men and 94.9% in women). Figures 2 and 3 also reveal a proximity between the recommended number of daily steps and R_y (78.4% in men and 87.4% in women).

#### Ecoproducts

Women who were 50 or older (94.7%), employed (85.7%), married (89.5%) with at least 2 children (94.9%) and documented a greater connection with nature (88.1%) were those who valued buying eco-products the most. Men with a dog showed a higher purchase of environmentally friendly products and brands (84.2%). As observed in the centroid graphs illustrated in Figures 2 and 3, the STP_r variable also showed close to EC_y in both genders, accounting for 70.6% in men and 80.0% in women.

#### Seasonal/Local

Regarding the purchase of seasonal and local products, and as observed in the upper right quadrant of Figure 3, it was women who manifested a high connection to the natural environment (91.0%), in the 33-49 age group (95.2%), and who performed at least 10 000 steps per day (90.5%) who exhibited a preference for purchasing seasonal and locally grown products.

#### Green travel

Men who met recommended levels of MVPA (93.8%) took more ecological travel. In the upper right quadrant of Figure 2 and regarding this PEB, we found that those who visited nature 2 to 3 times a week (89.7%), and who perform at least 10 000 steps daily (86.3%), showed a preference for walking and bicycling for their commuting.

#### Volunteering

Men who visited natural spaces 2 to 3 times a week (65.5.%), who achieved recommended levels of MVPA (53.1%) and number of daily steps (51.0%), and who had at least one child (55.6%) volunteered to help care for the environment.

#### Encouragement

In males, be married (94.3%), higer CN (100%), have a dog (100%) and performed the number recommended of daily steps (88.2%) established an association with the “ Encouragement” behavior.

It should be emphasized that for the “Seasonal/Local” behavior and for the “Encouragement” behavior, in the male gender, the group of “no” presents only 8 observations, so its reading and interpretation should be cautious, not overestimating the associations identified.

## Discussion

This study sought to analyze the relationship between pro-environmental behaviors and visits to natural spaces, connection with nature and physical activity in both genders. The results showed that women who were more connected to nature showed a preference for purchasing environmentally friendly products and brands and for local and seasonal products. Men with higher levels of MVPA and who walked more preferred to use the bicycle to commute. On the other hand, those who visited nature more frequently expressed greater involvement in unpaid activities oriented towards preserving the environment.

### Pro-Environmental Behaviors: Gender Differences

Gender plays an important role in the adoption of pro-environmental behavior. Some authors, have found that women express a greater number of sustainable attitudes, concerns, and behavior than men.^27-29^ The role played by women within the family and community as caregivers and educators reflects values such as altruism, compassion, cooperation and empathy, making them active agents in the environmental conservation.^46^ In contrast, men tend to see nature as something to be used for their own benefit.^47,48^ According to Loarne-Lemaire, Bertrand, Razgallah, Maalaoui and Kallmuenzer,^49^ women are more vulnerable to climatic problems arising from social, economic and cultural factors, showing a greater sensitivity to environmental challenges. The study conducted by Norgaard and York^50^ revealed that women in government positions signed more international treaties aimed at reducing global warming than their male counterparts.

For some authors, the gender differences associated with the adoption of pro-environmental behaviors depend on the dimensions considered, with women expressing more PEBs in the private sphere (for example, waste recycling, purchase of eco-products, and energy saving), compared to men. However, PEBs in the public sphere (affiliation with environmental organizations, active participation in social movements and demonstrations, donating money or signing petitions) seem to be adopted equally by both genders.^4,51,52^

Similar to other studies,^4^ our study did not identify differences between men and women in the number of PEBs adopted. Perhaps if our study comprised a more equal number of men and women, this would contribute to different results.

### Pro-Environmental Behaviors: Relationship With Other Analyzed Variables

In our study, men who exhibited higher levels of total and moderate-vigorous PA adopted a greater number of PEBs. According to Cunningham, McCullough and Hohensee,^53^ more active people, particularly those who prefer to practice PA while in contact with nature, tend to be more concerned about the environment.

Men who owned dogs also adopted a greater number of PEBs. Several authors^11,54,55^ identified a positive association between these variables and did not differentiate them according to gender.

In women, we identified an association between demographic variables and PEB adoption, particularly age and occupational status. Age is one of the most explored demographic factors among studies in the field of environmental concern.^56-59^ However, studies addressing the relationships between these variables have shown contradictory results. Some authors^60-62^ have argued that younger people, compared to previous generations, are more concerned with the health of the planet, resulting from greater exposure to climate change. Other research shows that older individuals adopt a greater number of PEBs, also showing a greater connection with the natural environment.^63^ and pursuing pro-social goals grounded in active participation in their communities.^64^

Occupational status was also associated with the adoption of a greater number of PEBs, but only in women. However, we were unable to infer whether this relationship was positive or negative from our results, due to the statistical method used.

According to authors,^65,66^ employed individuals adopt more PEBs than the unemployed individuals, perhaps because environmental education and volunteering are usually conducted in the corporations/entities where they work. For Rydzewski,^67^ employees, trainees, or students are more willing to make sacrifices for the benefit of the natural environment compared to the unemployed or retired. In contrast, Meyer^68^ argues that for some dimensions of PEB the unemployed people may exhibit greater involvement with them.

### Private and Public Dimensions of Pro-environmental Behaviors

#### Recycling

This study identified a significant association between age and “recycling” behavior in both genders. Miafodzyeva and Brandt^69^ report that people between 36 and 65 years old are the ones who recycle the most, this behavior is a “social norm,” although it depends on the time available to do it and the knowledge related to the conversion process of the potentially useful products. Employed women also revealed a close relationship with PEB, which is in line with other studies.^70^ In contrast, according to Meyer,^68^ unemployed people are the ones who mostly adopt behaviors that require effort or time (eg, recycling, water and energy savings) and that do not require financial burden.

The relationship between proximity to recycling in married women was also identified. The relationship between marital status and PEB adoption has been explored in several studies,^66,71-74^ documenting that married individuals are more concerned with environmental preservation. However, these studies did not differentiate this behavior according to gender. The literature also documents that women with more children are most concerned about preserving the environment.^75^ In our study, this relationship was observed only in men. We consider that the research would have benefited from the collection of information regarding the age of the children and whether they lived at home with their parents, factors that would certainly influence the adoption of PEB by the parents.^75^

Our study identified a close relationship between the recommended number of daily steps and recycling in both genders, with household waste sorting requiring more physical involvement.

#### Ecoproducts

In the present study, men with dogs showed a preference for purchasing environmentally friendly products and brands. According to Chomey,^76^ pet ownership increases opportunities for observation and interaction and encourages literacy related to environmental conservation.

The preference for buying these environmentally friendly products was also documented by men who visited nature to 2-3 times a week; these results are similar to those identified in another investigation.^11^ According to the authors, visiting natural environments more frequently for recreational purposes increases PEB adoption, contributing to the acquisition of products that are beneficial to the environment and health.

The purchase of environment-friendly products and brands was also privileged by older women, employed, married, with at least two children and who revealed a greater connection with nature. According to Tighe,^77^ individuals aged over 34 show a greater preference for purchasing products with environmentally friendly packaging compared to individuals belonging to younger age groups.

Some authors^78,79^ have shown that the purchase of eco-products is positively correlated with buyers' income, and that their occupational status contributes to their purchase, which is usually more expensive than conventional products. In our study, being married also influenced the adoption of PEB, corroborating the results documented by Shao, Li, Aneye and Fang,^80^ encouraging the partner to jointly adopt PEB.

The association between the number of children and the purchase of eco-products may come from the concern of mothers regarding the health and growth of their children, leading them to adopt strict requirements in their choice of products and brands.^75,81^

As for the connection with nature, the relationship found in the present study has also been identified in other investigations,^82,83^ although the authors did not differentiate the results by gender. According to them, individuals who are more connected to nature consider buying environmentally friendly products in line with their personal values and contribute to more sustainable consumption.

In the present investigation, ecoproducts were purchased by men and women who performed at least 10 000 steps daily. These results are consistent with those identified in other studies that documented that the purchase of less processed, healthier, and more environmentally beneficial foods was valued by more active individuals.^84,85^

#### Seasonal/local

The purchase of local and seasonal products generates economic, environmental and social benefits, favoring more sustainable patterns of production and consumption. Purchasing these products helps preserve local farmland, contributing to halting the loss of biodiversity and improving ecosystem health.^86,87^

In our study, women who were more connected to the natural environment showed a preference for buying seasonal and locally grown products, which is similar to the results of other surveys of men and women.^11^ This association can be explained by the fact that the latter are usually the most involved in making the purchases for the home.^88,89^

The results also showed that women aged 33-49 years and more active (performing at least 10 000 steps daily) showed a greater proximity to the manifestation of PEB, and CN may have mediated the relationship between these variables and “seasonal/local” behavior.

#### Green travel

Men with higher levels of MVPA favored active modes of travel, such as walking or bicycling, and performed a greater number of daily steps. Even though there were no differences in PA levels between the two genders, they exhibited greater engagement with the “green travel” behavior. According to Sánchez, Isabel and González,^90^ social/parental responsibilities mean that women are usually more dependent than men on using cars for shopping and transporting children or other family members in their care. On the other hand, men value the use of bicycles for recreational purposes and as a means of transport to work, spending more time on their use.^91^ The results also showed that men who visited nature 2-3 times a week exhibited a positive relationship with PEB. According to some authors,^92-94^ natural environments promote the use of active modes of transport and encourage ecologically sustainable travel.

#### Volunteering

Men who visited nature to 2-3 times a week volunteered more to care for and protect the environment. Similar results have been identified in other studies,^5,11^ which showed that visiting nature encourages dedication to initiatives to protect, care for, and restore the environment.^95,96^ Environmental volunteering was also more evident in men exhibited higher levels of weekly MVPA and who performed a higher number of daily steps, corroborating the results documented by other authors and whose PA was assessed using questionnaires.^97,98^ In the research conducted by Librett, Yore, Buchner and Schmid^97^ Individuals who did environmental volunteering were 2.6 times more likely to achieve recommended levels of PA for health, compared to those who engaged in other social and community actions without financial return.

Our study also revealed that men without children or with only one child had a higher participation as volunteers in environmental protection projects. Individuals without children may have more free time to engage in these initiatives,^99^ but at the same time, the presence of children in the household may also help promote environmental volunteerism.^100^

#### Encouragement

In our study, men who were married, had a dog, and/or were more connected to nature played an active role in encouraging others to use resources more efficiently by eradicating environmentally damaging behavior. Of all the pro-environmental behaviors, “encouragement” is still understudied,^101^ and there are no published studies that analyze its relationship with dog ownership, employment status, and marital status.

Regarding the association of PEB with CN, the literature states that a greater connection to the natural environment increases the sense of responsibility and commitment to preserve it, encouraging communities to adopt activities that reinforce the conservation and protection of their resources and promote ecological sustainability.^11,15,17,102^

The present investigation also showed that men who were employed and who performed at least 10 000 steps a day exhibited a closer relationship with “encouragement,” but further exploratory studies are needed in this context.

To our knowledge, this study is the first to explore the relationship between physical activity levels and PEBs in adults. Another strength of the manuscript is the assessment of physical activity levels by accelerometry and the inclusion of a range of demographic variables, which are still under-explored in the field of environmental concerns. Likewise, the analysis conducted separately for each gender revealed to be crucial. The fact that we included a list of behaviors, comprising both private and public dimensions, also proved to be important.

There are several limitations that influence the interpretation of these findings. First, our sample has significantly more women than men, which may have biased the results on gender differences. Second, as the study was cross-sectional cannot establish a cause-and-effect relationship or analyze behavior over a period of time. Third, the participants were not randomly selected, which may not be representative of the general population. Since our sample lives in areas with a high amount of green space, it could also be pointed out as a limitation, since this greater proximity to natural environments could be reflected in higher levels of PA. Other key limitation was that we did not detail the type of nature the respondents were visiting; hence, we could clearly see whether it was, for example public (park, forest, etc.) or private nature (garden and green terrace/balcony). Finally, the period when data collection was conducted (during winter, which is cold and rainy, and simultaneously during the COVID-19 pandemic where various movement restrictions were imposed) may have influenced not only levels of physical activity, but also visits to natural spaces, connection with nature, and the adoption of PEBs.

This study suggest several possibilities for future research. Analysis of the demographic variables proved to be of considerable importance, confirming the need for inclusion in future investigations. Much more work will also be needed to determine the influence of the coverage of green space in the area of residence on the adoption of environmentally friendly behavior. Further research combining simultaneous measurements of accelerometery and other participant wearables (eg, geo-locators) could provide more accurate information about physical activity levels in natural environments. Furthermore, it would be interesting to assess the time that individuals spend when visiting nature because, according to White, Alcock, Grellier, Wheeler, Hartig, Warber, Bone, Depledge and Fleming,^7^ spending at least 120  minutes a week in nature is associated with good health and well-being. Future studies on the current topic are therefore recommended to collect information regarding the type of contact with nature: intentional, incidental, or indirect. Developing and implementing structured physical activity programs in natural environments that simultaneously encourage the adoption of active and sustainable lifestyles, while educating for environmental awareness, are also necessary.

The development of policies and practices that promote walking (eg, investing in footpaths), cycling (eg, investing in bicycle lanes), sport, active recreation, and play, as well as the planning and design of new and existing urban green spaces to increase their use and enjoyment, can become drivers for adopting pro-environmental behaviors. These measures may be reflected in more visits to nature and probably higher levels of physical activity, factors that in our study were related to the adoption of sustainable behaviors. This is an important practical consideration for planners and managers particularly for natural environments that serve communities. Protecting and investing in natural resources in order to maximize the health and sustainability benefits they provide could be an important insight for policy makers and planners. In addition, policies that improve accessibility and encourage people to get out into natural environments could also play a key role in increasing levels of physical activity and the adoption of pro-environmental behaviors.

In conclusion, we found that a greater connection to the natural environment motivated the purchase of eco-friendly products and brands and the consumption of local and seasonal products by the females. In turn, higher levels of MVPA were recorded in men who were positively involved with the behavior of “green travel”. An association between engaging in environmental volunteering and nature visits has also been documented in men.

**SO WHAT?**

**What is already known on this topic?**

Evidence suggests that visiting nature more frequently and a greater connection with the natural environment are promoters of environmental conservation and protection, encouraging the adoption of pro-environmental behaviors. However, little is known about the relationship between physical activity and PEBs and how these relationships are differentiated by gender.

**What does this article add?**

To our knowledge, our study is pioneering in investigating the relationship between physical activity levels and the adoption of pro-environmental behaviors in adults. Our results showed an association between engaging in the recommended amount of physical activity with pro-environmental behaviors including recycling, preference for purchasing seasonal and locally grown products, green travel and volunteering in the environment (in men), and encouraging others to be pro-environmental (in men).

**What are the implications for health promotion practice or research?**

Our results may be useful for urban planners, green space managers, and public health professionals involved in urban sustainability and health protection, as it can help them design effective environmental practices and policies that encourage visits to natural spaces and promote physical activity.

## Supplemental Material

Supplemental Material - Pro-Environmental Behaviors: Relationship With Nature Visits, Connectedness to Nature and Physical Activity Click here for additional data file. Supplemental Material for Pro-Environmental Behaviors: Relationship With Nature Visits, Connectedness to Nature and Physical Activity by Andreia Teixeira, Ronaldo Gabriel, José Martinho, Mário Santos, Aurélio Faria, Irene Oliveira and Helena Moreira in American Journal of Health Promotion

## References

[1] Lange F Dewitte S. Measuring pro-environmental behavior: Review and recommendations. J Environ Psychol. 2019;63:92-100. doi:10.1016/j.jenvp.2019.04.009.

[2] Byerly H Balmford A Ferraro P, et al. Nudging pro-environmental behavior: Evidence and opportunities. Front Ecol Environ. 2018;16(3):159-168. doi:10.1002/fee.1777.

[3] Steg L Vlek C. Encouraging pro-environmental behaviour: An integrative review and research agenda. J Environ Psychol. 2009;29(3):309-317. doi:10.1016/j.jenvp.2008.10.004.

[4] Hadler M Haller M. Global activism and nationally driven recycling: The influence of world society and national contexts on public and private environmental behavior. Int Sociol. 2011;26(3):315-345. doi:10.1177/0268580910392258.

[5] Martin L White M Hunt A Richardson M Pahl S Burt J. Nature contact, nature connectedness and associations with health, wellbeing and pro-environmental behaviours. J Environ Psychol. 2020;68:101389. doi:10.1016/j.jenvp.2020.101389.

[6] DeVille N Tomasso L Stoddard O, et al. Time spent in nature is associated with increased pro-environmental attitudes and behaviors. Int J Environ Res Public Health. 2021;18(14):7498. doi:10.3390/ijerph18147498. 34299948 PMC8305895

[7] White M Alcock I Grellier J, et al. Spending at least 120 minutes a week in nature is associated with good health and wellbeing. Sci Rep. 2019;9(1):7730. doi:10.1038/s41598-019-44097-3. 31197192 PMC6565732

[8] Labib S Lindley S Huck J. Spatial dimensions of the influence of urban green-blue spaces on human health: A systematic review. Environ Res. 2020;180:108869. doi:10.1016/j.envres.2019.108869. 31722804

[9] Bratman G Anderson C Berman M, et al. Nature and mental health: An ecosystem service perspective. Sci Adv. 2019;5(7):eaax0903. doi:10.1126/sciadv.aax0903. 31355340 PMC6656547

[10] Frumkin H Bratman G Breslow S, et al. Nature contact and human health: A research agenda. Environ Healt Perspect. 2017;125(7):075001. doi:10.1289/ehp1663. PMC5744722 28796634

[11] Alcock I White M Pahl S Duarte-Davidson R Fleming L. Associations between pro-environmental behaviour and neighbourhood nature, nature visit frequency and nature appreciation: Evidence from a nationally representative survey in England. Environ Int. 2020;136:105441. doi:10.1016/j.envint.2019.105441. 31927464

[12] Larson L Whiting J Green G. Exploring the influence of outdoor recreation participation on pro-environmental behaviour in a demographically diverse population. Local Environ. 2011;16(1):67-86. doi:10.1080/13549839.2010.548373.

[13] Lawrence E. Visitation to natural areas on campus and its relation to place identity and environmentally responsible behaviors. J Environ Educ. 2012;43(2):93-106. doi:10.1080/00958964.2011.604654.

[14] Byrka K Hartig T Kaiser F. Environmental attitude as a mediator of the relationship between psychological restoration in nature and self-reported ecological behavior. Psychol Rep. 2010;107(3):847-859. doi:10.2466/07.pr0.107.6.847-859. 21323143

[15] Mackay C Schmitt M. Do people who feel connected to nature do more to protect it? A meta-analysis. J Environ Psychol. 2019;65:101323. doi:10.1016/j.jenvp.2019.101323.

[16] Pritchard A Richardson M Sheffield D McEwan K. The relationship between nature connectedness and eudaimonic well-being: A meta-analysis. J Happiness Stud. 2020;21(3):1145-1167. doi:10.1007/s10902-019-00118-6.

[17] Whitburn J Linklater W Abrahamse W. Meta-analysis of human connection to nature and proenvironmental behavior. Conserv Biol. 2020;34(1):180-193. doi:10.1111/cobi.13381. 31251416 PMC7027494

[18] Mayer F Frantz C. The connectedness to nature scale: A measure of individuals’ feeling in community with nature. J Environ Psychol. 2004;24(4):503-515. doi:10.1016/j.jenvp.2004.10.001.

[19] Russell R Guerry A Balvanera P, et al. Humans and nature: How knowing and experiencing nature affect well-being. Annu Rev Environ Resour. 2013;38(1):473-502. doi:10.1146/annurev-environ-012312-110838.

[20] Abu-Omar K Gelius P Messing S. Physical activity promotion in the age of climate change. F1000Res. 2020;9:349. doi:10.12688/f1000research.23764.2. 33628429 PMC7883311

[21] Salvo D Garcia L Reis R, et al. Physical activity promotion and the United Nations sustainable development goals: Building synergies to maximize impact. J Phys Act Health. 2021;18(10):1163-1180. doi:10.1123/jpah.2021-0413. 34257157

[22] WHO. Global action plan on physical activity 2018–2030: More active people for a healthier world. world health organization. https://apps.who.int/iris/bitstream/handle/10665/272722/9789241514187-eng.pdf. Accessed November 16, 2021.

[23] Braun T Cottrell R Dierkes P. Fostering changes in attitude, knowledge and behavior: Demographic variation in environmental education effects. Environ Educ Res. 2018;24(6):899-920. doi:10.1080/13504622.2017.1343279.

[24] Alp E Ertepinar H Tekkaya C Yilmaz A. A statistical analysis of children’s environmental knowledge and attitudes in Turkey. Int Res Geogr Environ. 2006;15(3):210-223. doi:10.2167/irgee193.0.

[25] Liu T Geng L Ye L Zhou K. “Mother Nature” enhances connectedness to nature and pro-environmental behavior. J Environ Psychol. 2019;61:37-45. doi:10.1016/j.jenvp.2018.12.003.

[26] DGT. Portugal’s Official Administrative Map - CAOP2020 (Continente). Directorate-General for the Territory. http://mapas.dgterritorio.pt/ATOM-download/CAOP-Cont/Cont_AAD_CAOP2020.zip. Accessed May, 28, 2021.

[27] DGT. Land use and land cover map - 2018. Directorate-general for the territory upon request. http://mapas.dgterritorio.pt/DGT-ATOM-download/COS_Final/COS2018_v1/COS2018_v1.zip. Accessed May, 28, 2021.

[28] DGT. Simplified Land Occupation Map. 2020 Updated to March 2021. https://docs.google.com/forms/d/e/1FAIpQLScYsQItiKrXmZQEqLzxbBHjEejfqtPZZb7LQxB8fKOAsUD7yQ/viewform?usp=sf_link

[29] Aragonês J Olivos P Lima M Loureiro A. La actividade en la naturaleza y su relación com la conetividad y el bienestar. Paper presented in XXXIV Interamerican Congress of Psychology; July, 2013; Rio de Janeiro, Brazil.

[30] Arvidsson D Fridolfsson J Börjesson M. Measurement of physical activity in clinical practice using accelerometers. 10.1111/joim.12908. J Intern Med. 2019;286(2):137-153. doi:10.1111/joim.12908. 30993807

[31] Migueles J Cadenas-Sanchez C Ekelund U, et al. Accelerometer data collection and processing criteria to assess physical activity and other outcomes: A systematic review and practical considerations. Sports Med. 2017;47(9):1821-1845. doi:10.1007/s40279-017-0716-0. 28303543 PMC6231536

[32] Matthews C Hagströmer M Pober D Bowles H. Best practices for using physical activity monitors in population-based research. Med Sci Sports Exerc. 2012;44(1 Suppl 1):S68-S76. doi:10.1249/MSS.0b013e3182399e5b. 22157777 PMC3543867

[33] Choi L Ward S Schnelle J Buchowski M. Assessment of wear/nonwear time classification algorithms for triaxial accelerometer. Med Sci Sports Exerc. 2012;44(10):2009-2016. doi:10.1249/MSS.0b013e318258cb36. 22525772 PMC3443532

[34] Rhudy M Dreisbach S Moran M Ruggiero M Veerabhadrappa P. Cut points of the Actigraph GT9X for moderate and vigorous intensity physical activity at four different wear locations. J Sports Sci. 2019;38(5):503-510. doi:10.1080/02640414.2019.1707956. 31865845

[35] Bull F Al-Ansari S Biddle S, et al. World health organization 2020 guidelines on physical activity and sedentary behaviour. Br J Sports Med. 2020;54(24):1451-1462. doi:10.1136/bjsports-2020-102955. 33239350 PMC7719906

[36] Tudor-Locke C Bassett D. How many steps/day are enough? Sports Med. 2004;34(1):1-8. 14715035 10.2165/00007256-200434010-00001

[37] Tudor-Locke C Craig C Aoyagi Y, et al. How many steps/day are enough? For older adults and special populations. Int J Behav Nutr Phys Act. 2011;8:80. doi:10.1186/1479-5868-8-80. 21798044 PMC3169444

[38] Field A. Discovering Statistics Using IBM SPSS Statistics. 4th ed. Sage; 2013.

[39] Cohen L Holliday M. Statistics for Social Sciences. Harper & Row; 1982.

[40] SPSS SPSS Categories® 14.0. Prentice Hall; 2005.

[41] Torgler B García-Valiñas M. The determinants of individuals’ attitudes towards preventing environmental damage. Ecol Econ. 2007;63(2-3):536-552. doi:10.1016/j.ecolecon.2006.12.013.

[42] Boeve-de Pauw J Van Petegem P. The effect of flemish eco-schools on student environmental knowledge, attitudes, and affect. Int J Sci Educ. 2011;33(11):1513-1538. doi:10.1080/09500693.2010.540725.

[43] Oerke B Bogner F. Gender, age and subject matter: impact on teachers’ ecological values. Environmentalist. 2010;30(2):111-122. doi:10.1007/s10669-009-9250-4.

[44] Loarne-Lemaire S Bertrand G Razgallah M Maalaoui A Kallmuenzer A. Women in innovation processes as a solution to climate change: A systematic literature review and an agenda for future research. Technol Forecast Soc Change. 2021;164(3):120440. doi:10.1016/j.techfore.2020.120440.

[45] Norgaard K York R. Gender equality and state environmentalism. Gend Soc. 2005;19(4):506-522. doi:10.1177/0891243204273612.

[46] Downward P Hallmann K Rasciute S. Volunteering and leisure activity in the United Kingdom: A longitudinal analysis of males and females. Nonprofit Volunt Sect Q. 2020;49(4):757-775. doi:10.1177/0899764020901815.

[47] Trelohan M. Do women engage in pro-environmental behaviours in the public sphere due to social expectations? The effects of social norm-based persuasive messages. VOLUNTAS. 33, 134, 148, 2021; doi:10.1007/s11266-020-00303-9.

[48] Cunningham MCB Hohensee S. Physical activity and climate change attitudes. Clim Change. 2020;159(1):61-74. doi:10.1007/s10584-019-02635-y.

[49] Fang W Ng E Chang M. Physical outdoor activity versus indoor activity: Their influence on environmental behaviors. Int J Environ Res Publ Health 2017;14(7):797. doi:10.3390/ijerph14070797. PMC5551235 28714934

[50] Arendt F Matthes J. Nature documentaries, connectedness to nature, and pro-environmental behavior. Environ Commun. 2016;10(4):453-472. doi:10.1080/17524032.2014.993415.

[51] Grajal A Luebke J Clayton S, et al. The relationship between affective connections to animals and proenvironmental behaviors. Conserv Biol. 2016;31(2):322-330. doi:10.1111/cobi.12780. 27310833

[52] Xue B McMunn A. Gender differences in unpaid care work and psychological distress in the UK Covid-19 lockdown. PLoS One. 2021;16(3):e0247959. doi:10.1371/journal.pone.0247959. 33662014 PMC7932161

[53] Evertsson M Nermo M. Changing resources and the division of housework: A longitudinal study of swedish couples. Eur Sociol Rev. 2007;23(4):455-470. doi:10.1093/esr/jcm018.

[54] Okin G. Environmental impacts of food consumption by dogs and cats. PLoS One. 2017;12(8):e0181301. doi:10.1371/journal.pone.0181301. 28767700 PMC5540283

[55] Kachnič J Sasáková N Papajová I, et al. The risk to human health related to disposal of animal wastes to soil — microbiological and parasitical aspects. Helminthologia. 2013;50(3):147-154. doi:10.2478/s11687-013-0124-4.

[56] Adane L Muleta D. Survey on the usage of plastic bags, their disposal and adverse impacts on environment: A case study in Jimma city, southwestern Ethiopia. Toxicol Environ Health Sci. 2011;3(8):234-248. doi:10.5897/JTEHS.9000067.

[57] Aral Ö López-Sintas J. A comprehensive model to explain europeans’ environmental behaviors. Sustainability. 2020;12(10):4307. doi:10.3390/su12104307.

[58] Gifford R Nilsson A. Personal and social factors that influence pro-environmental concern and behaviour: A review. Int J Psychol. 2014;49:141-157. doi:10.1002/ijop.12034. 24821503

[59] Blankenberg A Alhusen H. On the determinants of pro-environmental behavior - A guide for further investigations. SSRN Electron J. 2018;doi:10.2139/ssrn.3186089.

[60] Smith M Kingston S. Demographic, attitudinal, and social factors that predict pro-environmental behavior. Sustain Clim Change. 2021;14(1):47-54. doi:10.1089/scc.2020.0063.

[61] Subiza-Pérez M Marina L Irizar A, et al. Who feels a greater environmental risk? Women, younger adults and pro-environmentally friendly people express higher concerns about a set of environmental exposures. Environ Res. 2020;181:108918. doi:10.1016/j.envres.2019.108918. 31759645

[62] López-Mosquera N Lera-López F Sánchez M. Key factors to explain recycling, car use and environmentally responsible purchase behaviors: A comparative perspective. Resour Conserv Recycl. 2015;99:29-39. doi:10.1016/j.resconrec.2015.03.007.

[63] Welsch H Kühling J. Pro-environmental behavior and rational consumer choice: Evidence from surveys of life satisfaction. J Econ Psychol. 2010;31(3):405-420. doi:10.1016/j.joep.2010.01.009.

[64] Wiernik B Ones D Dilchert S. Age and environmental sustainability: A meta-analysis. J Manag Psychol. 2013;28(7/8):826-856. doi:10.1108/JMP-07-2013-0221.

[65] Wang Y Hao F Liu Y. Pro-environmental behavior in an aging world: Evidence from 31 countries. Int J Environ Res Public Health. 2021;18(4):1748. doi:10.3390/ijerph18041748. 33670167 PMC7916887

[66] Chen X Peterson M Hull V Lu C Hong D Liu J. How perceived exposure to environmental harm influences environmental behavior in urban China. Ambio. 2013;42(1):52-60. doi:10.1007/s13280-012-0335-9. 22821144 PMC3547460

[67] Chen X Peterson M Hull V, et al. Effects of attitudinal and sociodemographic factors on pro-environmental behaviour in urban China. Environ Conserv. 2011;38(1):45-52. doi:10.1017/S037689291000086X.

[68] Rydzewski P. The implementation of sustainable development vs. environmental attitudes in international comparative studies. Probl Ekorozwoju. 2013;8(1):125-137.

[69] Meyer A. Is unemployment good for the environment? Resour Energy Econ. 2016;45:18-30. doi:10.1016/j.reseneeco.2016.04.001.

[70] Miafodzyeva S Brandt N. Recycling behaviour among householders: Synthesizing determinants via a meta-analysis. Waste Biomass Valorization. 2013;4(2):221-235. doi:10.1007/s12649-012-9144-4.

[71] Zen I Noor Z Yusuf R. The profiles of household solid waste recyclers and non-recyclers in Kuala Lumpur, Malaysia. Habitat Int. 2014;42:83-89. doi:10.1016/j.habitatint.2013.10.010.

[72] Bagheri D Mohseni R Mahdavi S. Association of socioeconomic status and pro-environmental behaviors in the citizens of Gorgan, Iran (2017). Jorjani Biomed J. 2018;6(1):33-43. doi:10.29252/jorjanibiomedj.6.1.33.

[73] Dupont D. Do children matter? An examination of gender differences in environmental valuation. Ecol Econ. 2004;49(3):273-286. doi:10.1016/j.ecolecon.2004.01.013.

[74] Fisher C Bashyal S Bachman B. Demographic impacts on environmentally friendly purchase behaviors. J Target Meas Anal Market. 2012;20(3):172-184. doi:10.1057/jt.2012.13.

[75] Patel Modi A Paul J. Pro-environmental behavior and socio-demographic factors in an emerging market. Asian J Bus Ethics. 2017;6(2):189-214. doi:10.1007/s13520-016-0071-5.

[76] Migheli M. Green purchasing: The effect of parenthood and gender. Environ Dev Sustain. 2021;23(7):10576-10600. doi:10.1007/s10668-020-01073-6.

[77] Witek L Kuźniar W. Green purchase behavior: The effectiveness of sociodemographic variables for explaining green purchases in emerging market. Sustainability. 2020;13(1):209. doi:10.3390/su13010209.

[78] Chomey A. Our Unconscious Bridge to Nature: The Role of Pets and Animal Views in a Person's Environmental Attitudes, Conservation Habits, and Scientific Knowledge [MsD Dissertation]. Florida, USA: Faculty of the College of Arts and Sciences Florida Gulf Coast University; 2014.

[79] Tighe D. Opinion on sustainable products in Europe 2018, by age group. 2020.

[80] Alotoum F Nimri R. Antecedents of environmental buying behavior: Case of the jordanian market. Int J Bus Manag. 2015;10(9):240-250. doi:10.5539/ijbm.v10n9p240.

[81] Awad T. Environmental segmentation alternatives: Buyers’ profiles and implications. J Islam Mark. 2011;2:55-73. doi:10.1108/17590831111115240.

[82] Shao J Li W Aneye C Fang W. Facilitating mechanism of green products purchasing with a premium price—Moderating by sustainability-related information. Corp Soc Responsib Environ Manag. 2021;n/a(n/a):1-15. doi:10.1002/csr.2229.

[83] Jaiswal J Bihari S. Role of connectedness to nature and perceived environmental responsibility on green purchase behaviour. Asian J Bus Res. 2020;10(3):65-84. doi:10.14707/ajbr.200091.

[84] Dong X Liu S Li H Yang Z Liang S Deng N. Love of nature as a mediator between connectedness to nature and sustainable consumption behavior. J Clean Prod. 2020;242(1):118451. doi:10.1016/j.jclepro.2019.118451.

[85] Perkins H. Measuring love and care for nature. J Environ Psychol. 2010;30(4):455-463. doi:10.1016/j.jenvp.2010.05.004.

[86] Nie C Zepeda L. Lifestyle segmentation of US food shoppers to examine organic and local food consumption. Appetite. 2011;57(1):28-37. doi:10.1016/j.appet.2011.03.012. 21477631

[87] Soroka A Wojciechowska-Solis J. Consumer motivation to buy organic food depends on lifestyle. Foods. 2019;8(11):581. doi:10.3390/foods8110581. 31744098 PMC6915680

[88] Cvijanović D Ignjatijević S Vapa Tankosić J Cvijanović V. Do local food products contribute to sustainable economic development? Sustainability. 2020;12(7):2847. doi:10.3390/su12072847.

[89] Hiroki S Garnevska E McLaren S. Consumer perceptions about local food in New Zealand, and the role of life cycle-based environmental sustainability. J Agric Environ Ethics. 2016;29(3):479-505. doi:10.1007/s10806-016-9616-9.

[90] Schneider D. Gender deviance and household work: The role of occupation. Am J Sociol. 2012;117(4):1029-1072. doi:10.1086/662649. 22594117

[91] Cerrato J Cifre E. Gender inequality in household chores and work-family conflict. Front Psychol. 2018;9(1330) doi:10.3389/fpsyg.2018.01330. 30123153 PMC6086200

[92] Sánchez O Isabel M González E. Travel patterns, regarding different activities: Work, studies, household responsibilities and leisure. Transp Res Proc. 2014;3:119-128. doi:10.1016/j.trpro.2014.10.097.

[93] Heesch K Sahlqvist S Garrard J. Gender differences in recreational and transport cycling: A cross-sectional mixed-methods comparison of cycling patterns, motivators, and constraints. Int J Behav Nutr Phys Act. 2012;9(1):106. doi:10.1186/1479-5868-9-106. 22958280 PMC3503594

[94] Zhang L Zhou S Kwan M Chen F Lin R. Impacts of individual daily greenspace exposure on health based on individual activity space and structural equation modeling. Int J Environ Res Public Health. 2018;15(10):2323. doi:10.3390/ijerph15102323. 30360440 PMC6210249

[95] Lu Y Sarkar C Xiao Y. The effect of street-level greenery on walking behavior: Evidence from Hong Kong. Soc Sci Med (1982). 2018;208:41-49. doi:10.1016/j.socscimed.2018.05.022. 29758477

[96] Shanahan D Franco L Lin B Gaston K Fuller R. The benefits of natural environments for physical activity. Sports Med. 2016;46(7):989-995. doi:10.1007/s40279-016-0502-4. 26886475

[97] Ryan R Kaplan R Grese R. Predicting volunteer commitment in environmental stewardship programmes. J Environ Plan Manag. 2001;44(5):629-648. doi:10.1080/09640560120079948.

[98] Green L. Understanding the contribution parks and green spaces can make to improving people’s lives.Reading: Greenspace; 2010.

[99] Librett J Yore M Buchner D Schmid T. Take pride in America’s health: Volunteering as a gateway to physical activity. Am J Health Educ. 2005;36(1):8-13. doi:10.1080/19325037.2005.10608149.

[100] Pillemer K Fuller-Rowell T Reid M Wells N. Environmental volunteering and health outcomes over a 20-year period. Gerontologist. 2010;50(5):594-602. doi:10.1093/geront/gnq007. 20172902 PMC2937248

[101] Hoye J McGowan K Twist S Reeves D. Environmental volunteering social research report. Australia: Vitoria State Government: Environment, Land, Water and Planning; 2020.

[102] Taniguchi H. Men’s and women’s volunteering: gender differences in the effects of employment and family characteristics. Nonprofit Volunt Sect Q. 2006;35(1):83-101. doi:10.1177/0899764005282481.

