# Supplementary material for: Pro-Environmental Behaviors: Relationship With Nature Visits, Connectedness to Nature and Physical Activity
Source: Am J Health Promot. 2022 Aug 11;37(1):12–29. doi: 10.1177/08901171221119089 (PMC9755701; doi:10.1177/08901171221119089)
Supplement: Supplemental Material - Pro-Environmental Behaviors: Relationship With Nature Visits, Connectedness to Nature and Physical Activity [file sj-pdf-1-ahp-10.1177_08901171221119089.pdf]

Table S1. Operationalization of demographic variables

	Measure	Item	Responses
Demographic data	Gender	Respondent's sex	Male (0); Female (1)
	Age	What was your age last birthday?	<33 years (1); 33-49 years (2); ≥ 50 years
	Marital status	Respondent's marital status	Married/cohabiting (1); Single (2); Separated/divorced/widowed (3)
	Number of children	How many children do you have?	0 or 1 child (1); 2 or more children (2)
	Education	What are your academic qualifications?	No Education (0); Elementary school (1); Middle school (2); High school (3); University (4); Post-graduation/master's/doctorate (5)
	Occupational status	What is your occupational situation?	Employee (1); Unemployed (2); Retired (3); Student (4); Other (5)
	Dog ownership	Do you have dogs that you usually walk?	No (1); Yes (2)

Table S2. Summary of variables of the three sets, which are incorporated in the non-linear canonical correlation analyses

Sets	Variables	Variable type	Categories	Category symbols
Pro-environmental behaviors 1	Recycling	Single nominal	No	R_n
			Yes	R_y
	Eco-products	Single nominal	No	EC_n
			Yes	EC_y
	Seasonal/local	Single nominal	No	SL_n
			Yes	SL_y
	Green travel	Single nominal	No	GT_n
			Yes	GT_y
	Volunteering	Single nominal	No	V_n
			Yes	V_y
	Encouragement	Single nominal	No	E_n
			Yes	E_y
Nature visits, connectedness to nature and physical activity 2	Nature visits (NV)	Ordinal	Never or once a week	NV_≤1
			2-3 times a week	NV_2-3
			≥ 4 times a week	NV_≥4
	Connectedness to nature (CN)	Single nominal	Low CN ≤ 3.71	CN_l
			High CN > 3.71	CN_h
	Moderate -vigorous physical activity (MVPA)	Single nominal	Low MVPA < 150 min/week	MVPA_l
			High MVPA ≥ 150 min/week	MVPA_h
	Steps/day	Single nominal	Not Recommended < 10 000 steps	STP_nr
			Recommended ≥ 10 000 steps	STP_r
Demographic variables 3	Age groups	Ordinal	<3 3 years	< 33y
			33-49 years	33-49y
			≥ 50 years	≥ 50y
	Marital status	Ordinal	Single	MS_s
			Married/cohabiting	MS_m
			Separated/divorced/widowed	MS_sdw
	Number of children	Single nominal	0-1 child	CH_0-1
			≥ 2 children	CH_≥ 2
	Occupational status	Ordinal	Employee	OS_e
			Unemployed	OS_un
			Retired	OS_r
			Student	OS_s
			Other	OS_o
	Owns a dog	Single nominal	No	DOG_n
			Yes	DOG_y

Table S3. Percentages of dimensions 2 and 3 for men

**PEB** *MEN*			**Recycling**		**Eco-products**		**Green travel**		**Volunteering**		**Encouragement**	
		n	No	Yes	No	Yes	No	Yes	No	Yes	No	Yes
**Age**	< 33y	22	40.9%	59.1%	27.3%	72.7%	9.1%	90.9%	40.9%	59.1%	22.7%	77.3%
	33-49y	21	19.0%	**81.0%**	33.3%	66.7%	28.6%	71.4%	47.6%	52.4%	9.5%	**90.5%**
	≥ 50 y	18	16.7%	**83.3%**	44.4%	55.6%	16.7%	83.3%	66.7%	33.3%	5.6%	**94.4%**
**Marital status**	MS_s	26	34.6%	65.4%	26.9%	73.1%	15.4%	84.6%	50.0%	50.0%	23.1%	76.9%
	MS_m	35	20.0%	80.0%	40.0%	60.0%	20.0%	80.0%	51.4%	48.6%	5.7%	**94.3%**
**Number of children**	CH_0-1	36	33.3%	66.7%	27.8%	72.2%	19.4%	80.6%	44.4%	**55.6%**	16.7%	83.3%
	CH_≥ 2	25	16.0%	**84.0%**	44.0%	56.0%	16.0%	84.0%	60.0%	40.0%	8.0%	92.0%
**Occupational status**	OS_e	40	17.5%	82.5%	37.5%	62.5%	20.0%	80.0%	52.5%	47.5%	10.0%	**90.0%**
	OS_un	2	0.0%	100.0%	50.0%	50.0%	50.0%	50.0%	0.0%	100.0%	0.0%	100.0%
	OS_s	17	47.1%	52.9%	29.4%	70.6%	11.8%	88.2%	52.9%	47.1%	23.5%	76.5%
	OS_o	2	50.0%	50.0%	0.0%	100.0%	0.0%	100.0%	50.0%	50.0%	0.0%	100.0%
**Dog ownership**	DOG_n	42	28.6%	71.4%	42.9%	57.1%	19.0%	81.0%	54.8%	45.2%	19.0%	81.0%
	DOG_y	19	21.1%	78.9%	15.8%	**84.2%**	15.8%	84.2%	42.1%	57.9%	0.0%	**100.0%**
**Nature visits**	NV_≤ 1	30	26.7%	73.3%	43.3%	56.7%	23.3%	76.7%	66.7%	33.3%	16.7%	83.3%
	NV_2-3	29	27.6%	72.4%	27.6%	**72.4%**	10.3%	**89.7%**	34.5%	**65.5%**	10.3%	89.7%
	NV_≥ 4	2	0.0%	100.0%	0.0%	100.0%	50.0%	50.0%	50.0%	50.0%	0.0%	100.0%
**Connectedness to nature**	CN_l	32	31.3%	68.8%	43.8%	56.3%	21.9%	78.1%	56.3%	43.8%	25.0%	75.0%
	CN_h	29	20.7%	79.3%	24.1%	75.9%	13.8%	86.2%	44.8%	55.2%	0.0%	**100.0%**
**Moderate-vigorous PA**	MVPA_l	29	27.6%	72.4%	44.8%	55.2%	31.0%	69.0%	55.2%	44.8%	10.3%	89.7%
	MVPA_h	32	25.0%	75.0%	25.0%	75.0%	6.3%	**93.8%**	46.9%	**53.1%**	15.6%	84.4%
**Steps/day**	STP_nr	10	50.0%	50.0%	60.0%	40.0%	40.0%	60.0%	60.0%	40.0%	20.0%	80.0%
	STP_r	51	21.6%	**78.4%**	29.4%	**70.6%**	13.7%	**86.3%**	49.0%	**51.0%**	11.8%	**88.2%**

n - number of elements by group

Table S4. Percentages of dimensions 2 and 3 for women

**PEB**<br>*WOMEN*			**Recycling**		**Eco-products**		**Seasonal/Local**	
		n	No	Yes	No	Yes	No	Yes
**Age**	<33 y	53	26.4%	73.6%	37.7%	62.3%	11.3%	88.7%
	33-49y	42	7.1%	**92.9%**	16.7%	83.3%	4.8%	**95.2%**
	≥50 y	38	5.3%	**94.7%**	5.3%	**94.7%**	7.9%	92.1%
**Marital status**	MS_m	57	7.0%	93.0%	10.5%	**89.5%**	8.8%	91.2%
	MS_s	63	23.8%	76.2%	34.9%	65.1%	7.9%	92.1%
	MS_sdw	13	0.0%	100.0%	7.7%	92.3%	7.7%	92.3%
**Number of children**	CH_0-1	94	18.1%	81.9%	28.7%	71.3%	7.4%	92.6%
	CH_≥ 2	39	5.1%	**94.9%**	5.1%	**94.9%**	10.3%	89.7%
**Occupational status**	OS_e	84	83.3%	**91.7%**	14.3%	**85.7%**	7.1%	92.9%
	OS_un	2	50.0%	50.0%	50.0%	50.0%	0.0%	100.0%
	OS_r	3	0.0%	100.0%	0.0%	100.0%	0.0%	100.0%
	OS_s	38	26.3%	73.7%	42.1%	57.9%	13.2%	86.8%
	OS_o	6	16.7%	83.3%	0.0%	100.0%	0.0%	100.0%
**Dog ownership**	DOG_n	93	9.7%	**90.3%**	20.4%	79.65%	7.5%	92.5%
	DOG_y	40	25.0%	75.0%	25.0%	75.0%	10.0%	90.0%
**Nature visits**	NV_≤ 1	96	15.6%	84.4%	26.0%	74.0%	8.3%	91.7%
	NV_2-3	30	10.0%	90.0%	6.7%	93.3%	6.7%	93.3%
	NV_≥ 4	7	14.3%	85.7%	28.6%	71.4%	14.3%	85.7%
**Connectedness to nature**	CN_l	66	19.7%	80.3%	31.8%	68.2%	7.6%	92.4%
	CN_h	67	9.0%	**91.0%**	11.9%	**88.1%**	9.0%	**91.0%**
**Moderate-vigorous PA**	MVPA_l	67	16.4%	83.6%	19.4%	80.6%	6.0%	94.0%
	MVPA_h	66	12.1%	87.9%	24.2%	75.8%	10.6%	89.4%
**Steps/day**	STP_nr	38	18.4%	81.6%	26.3%	73.7%	5.3%	94.7%
	STP_r	95	12.6%	**87.4%**	20.0%	**80.0%**	9.5%	**90.5%**

n - number of elements by group
